# Comment on: a novel transition clinic structure for adolescent and young adult patients with childhood onset rheumatic disease improves transition outcomes

**DOI:** 10.1186/s12969-022-00718-2

**Published:** 2022-08-09

**Authors:** Mislav Radić

**Affiliations:** Division of Rheumatology and Clinical Immunology, Center of Excellence for Systemic Sclerosis in Croatia, University Hospital Split, School of Medicine, Split, Croatia

Dear Editor,

The article in Pediatric Rheumatology by Overbury et al. [1] proposing a transition clinic named ACCORD (Adult Center for Childhood Onset Rheumatic Disease) that integrates internal medicine - pediatrics trained adult rheumatologist in a pediatric rheumatology clinic is a very interesting novel concept. Successful transition of all indicated adolescents and young adults (AYA) with childhood onset rheumatic disease (CORD) to adult rheumatologist with a median time between last transition clinic visit and first adult clinic visit of just 5.1 months ensured continuity of medical care [1] and could potentially increase the likelihood of positive outcomes. Although European and American societies have recognized the importance of health care transition (HCT) and have developed international recommendations and standards for transitional care, universal implementation is still not realized. There is growing evidence of benefits of HCT [1–4], however there is no consensus on the optimal model since there is no systematic approach to evaluating and reporting the effectiveness of implemented programs across the globe. Creating appropriate questionnaires and their validation in order to monitor the activity and severity of the disease would significantly improve the quality of transition clinics.

Hence, a question: Has the time come for all relevant societies, like the efforts of Assessment of Spondyloarthritis International Society (ASAS)/European League Against Rheumatism (EULAR) in axial spondyloarthritis [5], to join together to develop recommendations for the care of this vulnerable group of patients?

The ACCORD clinic bridges the gap between pediatric and adult rheumatology, and it might be the right way forward in the care of AYA patients. Furthermore, ACCORD clinic is a step toward a life course management approach in rheumatology enabling trained rheumatologist in this field to manage transitional clinic. Multidisciplinary treatment (MDT) approach to the treatment of AYA patients with CORD involves a pediatric and adult rheumatologist requiring coordination of availability of experts. Another very valid question in MDT approach is: with whom lies the final responsibility and choice of treatment. Ultimately, this should be standardized in the same way as treatment recommendations. Furthermore, structured HCT process for youth with special health care needs has showed improvements in adherence to care, disease-specific measures, quality of life, self-care skills, satisfaction with care, health care utilization, and HCT process of care [6]. We have witnessed recent advances in rheumatology research and approval of wide range of disease-modifying anti-rheumatic drugs (DMARDs), biologic (bDMARDs) and targeted synthetic (tsDMARDs). However, not all bDMARDs/tsDMARDs are approved for adolescents limiting their treatment options. The ACCORD clinic has the possibility to take this aspect into consideration as well and improve access to appropriate therapy. Finally, HCT process could be a determining factor to prevent hospital admission rates, surgeries needed and adult clinic attendance rates [7].
